# Validity and Reliability of a Chinese-Language Instrument for Continuous Assessment of Exercise Stages of Change in Adults

**DOI:** 10.1097/jnr.0000000000000310

**Published:** 2019-07-16

**Authors:** Shih-Hui Chen, Hsiang-Ru Lai, Su-Ru Chen, Pi-Chu Lin, Kuei-Ru Chou, Pi-Hsia Lee

**Affiliations:** 1 MSN, RN, School of Nursing, College of Nursing, Taipei Medical University; 2 PhD, Professor, Department of Health Promotion and Health Education, National Taiwan Normal University; 3 PhD, RN, Associate Professor, Post-Baccalaureate Program in Nursing and School of Nursing, College of Nursing, Taipei Medical University; 4 EdD, RN, Professor, Master Program in Long-Term Care, College of Nursing, Taipei Medical University; 5 PhD, RN, Professor, School of Nursing, College of Nursing, Taipei Medical University; Department of Nursing, Shuang Ho Hospital, Taipei Medical University; and Psychiatric Research Center, Taipei Medical University Hospital; 6 EdD, RN, Professor, School of Nursing, College of Nursing, Taipei Medical University.

**Keywords:** instrument development, psychometric analysis, validity, reliability, exercise stages of change, continuous measurement

## Abstract

**Background:**

Single- and five-item measures have been used prevalently to assess exercise stages of change. Few studies have investigated the development of instruments that are able to continuously measure exercise stages of change and have conducted associated psychometric analyses.

**Purpose:**

This study aimed to translate the original, English-language version of the University of Rhode Island Change Assessment-Exercise 2 (URICA-E2), a continuous exercise stages of change assessment instrument, into Chinese and then to test the validity and reliability of the translated version.

**Methods:**

A cross-sectional descriptive study was conducted. Participants consisted of 325 adults from Taipei, Taiwan. The URICA-E2 was translated into Chinese using a standardized procedure. Psychometric analyses, including validity, reliability, and cluster analysis, of the Chinese-version instrument were conducted.

**Results:**

The content validity index was .987. Confirmatory factor analysis confirmed that the overall model fit was standardized, as the factor loadings of all of the items and the composite reliability and average variance extracted for the six exercise stages of change satisfied the convergent validity criteria. The average variance extracted for each construct of the stages of behavior change satisfied the discriminatory validity criteria. Values of Cronbach's α for the six exercise stages ranged from .80 to .94. The intraclass correlation coefficients for test–retest reliability after 2 weeks ranged between .74 and .87.

**Conclusions:**

The Chinese-language version of the URICA-E2 developed in this study exhibited excellent validity and reliability. This instrument may be used by healthcare professionals and the academic community to effectively and continuously measure the intentions and attitudes of adults at various exercise stages of change and to guide the provision of appropriate interventions.

## Introduction

Physical inactivity has risen to become the fourth leading risk factor for mortality worldwide and is now the primary risk factor for noncommunicable diseases and the cause of 3.2 million deaths annually (World Health Organization, 2018a). Therefore, enhancing physical activity among the populous, reducing the prevalence of noncommunicable diseases, and improving public health are important goals of health departments and healthcare personnel in various countries.

Among the numerous advantages of regular physical activity include weight control; reduced risks of cardiovascular disease, Type II diabetes, metabolic syndrome, and various cancers; stronger bones and muscles; enhanced mental health and emotional well-being; improved activities of daily living in elderly people; lower incidences of falls in elderly people; and increased longevity (Centers for Disease Control and Prevention, 2015). The World Health Organization (2018b) recommends that healthy adults undertake moderate-intensity exercise for > 150 minutes per week to develop and maintain their cardiopulmonary, musculoskeletal, and neuromotor fitness. Achieving the recommended level of physical activity is a goal toward which everyone should strive to promote physical and mental health.

The results of one recent investigation suggests that 33.4% of Taiwanese ≥ 13 years old exercise regularly (Health Promotion Administration, Ministry of Health and Welfare, Taiwan, ROC, 2016). This percentage is much lower than the percentages of adults who meet the physical activity recommendations in England and the United States (British Heart Foundation, 2015; Centers for Disease Control and Prevention, 2017). Over the past decade, health departments in Taiwan have formulated policies and allotted substantial resources to assist people to establish regular exercise habits. In addition, accurate behavioral assessment instruments are essential for examining people's exercise behaviors. Therefore, healthcare personnel should work to develop these instruments.

The transtheoretical model developed by Prochaska and DiClemente (1982) posits precontemplation, contemplation, preparation, action, and maintenance as the five stages of behavioral change. In terms of exercise behaviors, these stages of change are known as the “exercise stages of change.” Single- and five-item measures are two typical methods used to measure the exercise stages of change. Single-item measures employ a question with five choices, enabling the participant to select the choice that most closely resembles his or her current exercise stage of change (Marcus, Selby, Niaura, & Rossi, 1992). In contrast, the five-item measure contains five yes/no questions, and the various exercise stages of change are determined according to the participant's responses to these questions (Marcus & Simkin, 1993).

Reed (1995) reported that exercise is a type of continuous, cyclic behavior. Thus, identifying the current exercise stage of an individual and comprehending the unique characteristics of each stage are crucial to examining and understanding exercise behavior. The single- and five-item measures offer only a simple classification of exercise behaviors, and neither is designed to identify subsequent exercise intentions at the precontemplation, contemplation, and preparation stages or to ascertain previous exercise behaviors at the action and maintenance stages. The University of Rhode Island Change Assessment-Exercise 2 (URICA-E2) instrument, published by the university's Cancer Prevention Research Center, adopts a continuous measurement approach to assessing exercise stages of change. Compared with the single-item measure, which assesses only one stage of change, the URICA-E2 may be used to collect data from individuals on the six exercise stages of change (precontemplation-nonbeliever [PCNB], precontemplation-believer [PCB], contemplation, preparation, action, and maintenance). In addition, the URICA-E2 assumes a typical dynamic process in which exercise behaviors shift back and forth between the various exercise stages, which reflects how people develop complex attitudes and a mentality toward exercise at each point in time. Moreover, the continuous measurement approach is superior to the five-item measure because the former dynamically measures all six exercise stages of change. Furthermore, the URICA-E2 is based on the transtheoretical model for assessing stages of behavioral change (Lerdal et al., 2009; Reed, 1993).

The URICA-E2 is beneficial for integrating behavioral and cognitive constructs, because it clearly defines the constructs in each exercise stage. In addition, the URICA-E2 may be applied to cluster analyses to describe the typologies or profiles of people in the midst of change. Lerdal et al. (2009) recruited 198 undergraduate nursing students as research participants to develop a Norwegian version of the URICA-E2 and verified that this version may be used to accurately assess the extent of behavioral changes at various exercise stages. Therefore, both the English and Norwegian versions of the URICA-E2 are valid tools to assess people's views on exercise and to guide the development of efficacious exercise plans. However, the single- and five-item measures are the only instruments currently available in Taiwan to examine exercise stages of change. Therefore, the purpose of this study was to translate the English-language version of the URICA-E2 into Chinese and then test the validity and reliability of the translated instrument.

## Methods

### Design and Participants

A cross-sectional descriptive design was adopted. The English-language version of the URICA-E2 was translated into Chinese using a standardized procedure. According to the Brislin (1986) translation model and the recommendations of Beaton, Bombardier, Guillemin, and Ferraz (2000), the translation process used in this study included the following steps: (a) forward translation using two bilingual translators (one with a medical background and one with a master's degree in English) who separately translated the English-language version of the URICA-E2 into traditional Chinese, (b) synthesis of the translations: A person with a PhD degree in nursing and a Taiwan-based doctoral student from the United States who was proficient in Chinese integrated the two forward-translation copies into one copy of the C-URICA-E2, and (c) backward translation: A bilingual translator who had not used or read the original, English version of the URICA-E2 translated the C-URICA-E2 back into English. This translated English version of the URICA-E2 was compared with the original English version of the URICA-E2 to ensure that the concepts and content of the Chinese version were consistent with those of the original English version.

After the Chinese translation of the URICA-E2 (C-URICA-E2) was completed, six experts from the sports, nursing, health education, and public health fields were invited to examine the content validity as well as the meanings and appropriateness of the items in the questionnaire. Both content validity and construct validity were used to examine the validity of the instrument. Cronbach's α values and intraclass correlation coefficients (ICCs) were calculated to assess the reliability of the C-URICA-E2 (Koo & Li, 2016).

According to Comrey and Lee (1992), the minimum sample size required for a confirmatory factor analysis (CFA) is 300. A convenience sampling was adopted in this study. Volunteers aged 20–64 years were recruited at supermarkets (Carrefour, Amart, and RT-Mart) in each of the 12 administrative regions of Taipei City. Six hundred twenty-five eligible people were approached, and 325 agreed to participate. The total number of participants from each administrative region ranged between 13 and 36.

### Instrument

The original, English version of the URICA-E2 was developed by Reed (1993), who also performed a CFA that verified that the URICA-E2 exhibited excellent construct validity. The original, English-language version of the URICA-E2 comprises 24 items that measure the following six stages of change. Each item is scored from 1 = *strongly disagree* to 5 = *strongly agree*.

- PCNB: A person does not understand or intend to understand exercise behavior and therefore has no motivation to exercise and does not plan to undertake exercise.
- PCB: A person is aware of exercise behavior and has attempted to change his or her exercise behavior; however, because of external factors, he or she fails to change and thus does not intend to act.
- Contemplation: A person intends to undertake exercise within 6 months and is aware that he or she must change his or her exercise behavior but sometimes has contradictory feelings about this change.
- Preparation: A person will undertake exercise within 1 month; at this stage, he or she will participate in programs or activities for changing exercise behavior.
- Action: A person has undertaken exercise, but his or her exercise behavior has lasted for < 6 months; in other words, this person has modified his or her lifestyle within the past 6 months.
- Maintenance: A person has regularly undertaken exercise for ≥ 6 months.

### Data Collection and Ethical Considerations

After this study was approved by the university institutional review board (No. 201302020), a trained graduate student began in May 2013 to recruit potential participants at supermarkets. All of the potential participants were informed of the research purposes, procedures, and their rights and interests and were required to provide written consent before participation. The questionnaire was completed anonymously in 10–15 minutes. Each participant received a small incentive gift upon returning the completed questionnaire.

### Data Analysis

SPSS 19.0 for Windows (IBM Statistics, Armonk, NY, USA) and AMOS 17.0 for Windows (SPSS, Chicago, IL, USA) were employed in data analysis. A CFA was performed to examine the quality of the overall model and the construct validity (the convergent validity and discriminatory validity) of the instrument. In the CFA, a chi-square test resulting in *p* > .05 and a “chi-square to degrees of freedom” ratio or χ^2^/*df* of < 3 indicates that the model has a favorable goodness of fit. A goodness of fit index (GFI) and an adjusted GFI of > .9 as well as a root mean square error of approximation and a standardized root mean square residual of < .08 imply that these fit indices effectively explain the observed data. Moreover, if the normed fit index (NFI), non-NFI, and comparative fit index (CFI) are all > .9, then the observed data do not differ from the model-estimated data (Schreiber, Nora, Stage, Barlow, & King, 2006).

According to Fornell and Larcker (1981), convergent validity must fulfill the following criteria: The factor loading of an observed variable must be > .5, the composite reliability (CR) must be > .6, and the average variance extracted (AVE) for each latent variable must be > .5. According to Hair, Black, Babin, and Anderson (2014), at least 75% of all constructs must have a square root AVE value greater than the correlation coefficients between the constructs to show discriminatory validity.

A corrected item–total correlation analysis was performed to determine the utility of each questionnaire item according to the selection criterion of Pallant (2013; i.e., a coefficient value of ≥ .3). Internal consistency reliability was analyzed for the constructs of each concept, and the selection criterion was set at a Cronbach's α value of ≥ .7 (Pallant, 2013). Test–retest reliability was evaluated using ICCs, with values between .5 and .74 indicating moderate reliability and between .75 and .90 indicating good reliability (Portney & Watkins, 2015).

Cluster analysis was conducted using the *k*-means method to see if participants could appropriately be grouped into the six categories. The average scores for the six exercise stages of change were first converted into *Z* scores and then into *T* scores (Lerdal et al., 2009; Reed, 1995).

## Results

### Participant Characteristics

The 325 adults who participated in this study averaged 42.63 years old (*SD* = 12.83, range: 20–64 years). The percentage of women (61.5%) was higher than that of men. Most participants were married (49.5%) and had a bachelor's degree or higher (53.5%), 40.9% reported their health status as good or very good, and 69.5% reported having no chronic disease.

### Validity Analysis

The CVI for the C-URICA-E2 instrument was .987, indicating that the instrument had excellent content validity.

This study adopted a CFA to examine model fit, with results including χ^2^ = 539.60 (*df* = 237, *p* < .001), χ^2^/*df* = 2.28, GFI = .88, adjusted GFI = .85, root mean square error of approximation = .06, standardized root mean square residual = .06, NFI = .91, non-NFI = .94, and CFI = .95. All of the indices met the criteria for fitness with the exception of χ^2^ (*p* < .001), indicating a favorable model fit (i.e., the external quality of a model). Therefore, the C-URICA-E2 exhibited high construct validity.

**Figure 1.**
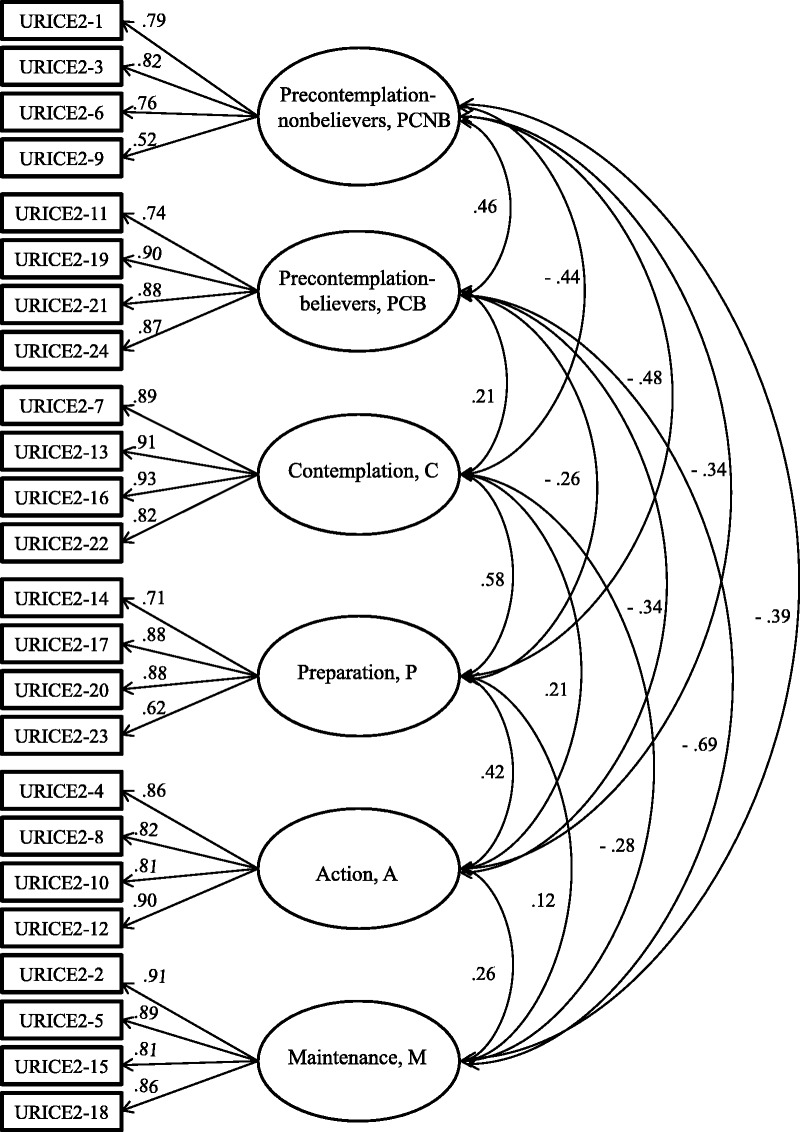
Confirmatory factor analysis model of the Chinese-language version of the University of Rhode Island Change Assessment-Exercise 2 (URICA-E2).

According to the standardized estimates of the CFA model (Figure 1), the standardized factor loadings were not > 1, indicating that no unreasonable parameters existed. Hair et al. (2014) suggested that a standardized factor loading must be > .7 and that the individual item reliability for an observed variable (square of the factor loading) must be > .5. In this study, the standardized factor loadings of items in each construct of the six exercise stages ranged between .71 and .93. Thus, the explanatory power of the items for each construct was > 50%, indicating that the C-URICA-E2 exhibited a strong-to-moderate association for all of the item–factor relationships except for the ninth and 23rd items, which exhibited weak associations (λ = .52 and .62, respectively).

The standardized factor loading for the convergent validity was higher than the suggested value of .5, the CR was > .6, and the square roots of AVE values for the variables were all > .5 (Table 1). Therefore, the constructs of the six exercise stages satisfied the criteria for convergent validity, indicating that the internal quality of the measurement model is excellent.

**TABLE 1.**
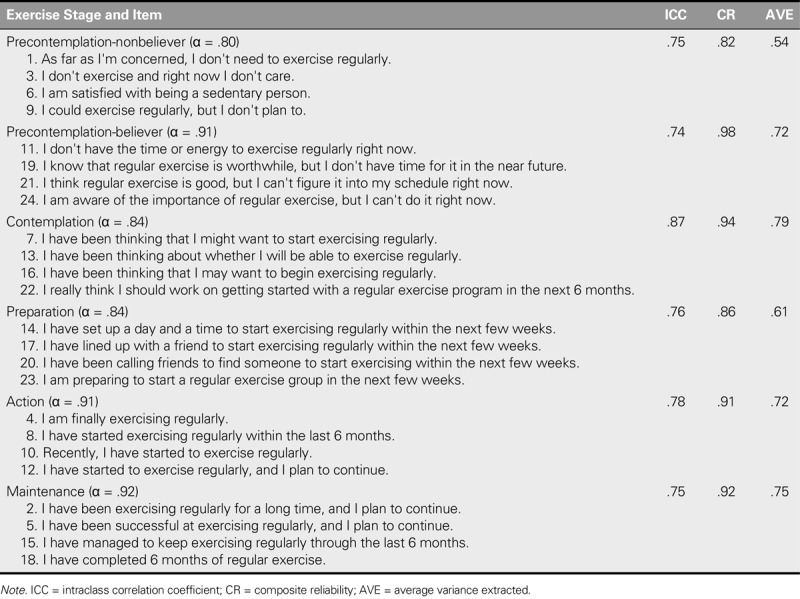
Convergent Validity and Reliability Analysis

The square roots of AVE values ranged from .73 to .89 and were greater than the correlation coefficients estimate on the off-diagonal in the matrix (−.69 to .58), indicating that the C-URICA-E2 exhibits high discriminatory validity (Table 2).

**TABLE 2.**
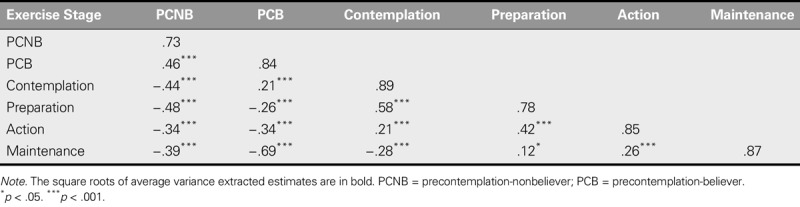
Assessment of Discriminatory Validity (*N* = 325)

### Reliability Analysis

The corrected item–total correlation coefficients of the C-URICA-E2 ranged between .32 and .87, indicating that all of the items are appropriate. Furthermore, Cronbach's α values for the six constructs ranged from .80 to .92, indicating that the instrument has high internal consistency (Table 1).

In this study, 40 adults were initially selected and retested after 2 weeks. ICCs for the C-URICA-E2 ranged from .74 to .87, indicating that the instrument is highly stable (Table 1).

### Cluster Analysis

Cluster analysis showed that six categories were generated, and the stage of “exercise” had the highest *T* score of all of the clusters. For the first cluster, preparation earned the highest *T* score (62.8). Thus, participants in Cluster 1 were in the exercise stage of preparation. Participants in Clusters 2–6 were, respectively, in the stages of contemplation, PCNB, maintenance, action, and PCB. The distribution of participants among the six exercise stages of change was 54 (16.6%) in PCNB, 19 (5.8%) in PCB, 110 (33.8%) in contemplation, 59 (18.2%) in preparation, 47 (14.5%) in action, and 36 (11.1%) in maintenance (Table 3).

**TABLE 3.**
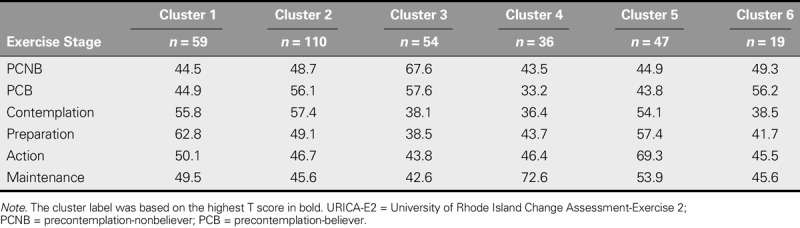
*T* Scores of the Constructs in Various Clusters of the Chinese-Language Version of the URICA-E2 (*N* = 325)

## Discussion

This study performed a CFA to assess the construct validity of the C-URICA-E2. The indices of model fit were χ^2^ = 539.59, *df* = 237, GFI = .88, and CFI = .95. Indices of the model fit of the original, English-language version of the URICA-E2 were previously identified as χ^2^ = 852.30, *df* = 237, GFI = .82, and CFI = .89. These results imply that the Chinese and English versions of this instrument have similar model fits. Therefore, both versions of the URICA-E2 exhibit favorable overall model fit and construct validity. The χ^2^ values for the two URICA-E2 versions did not fulfill the CFA criterion, which may be because of the influence of sample size on the χ^2^ test. In particular, an overall model with a large sample size can be easily rejected. Although the χ^2^ test did not satisfy the requirement of a low value, the results were not affected (Kline, 2016).

According to the CFA of the Chinese- and English-language versions of the URICA-E2, the factor loadings of the items for the constructs of the six exercise stages ranged from .52 to .93 and .54 to .95, respectively. The CFA revealed a strong-to-moderate association for item–factor relationships in the URICA-E2, indicating that the items are valid for measuring latent variables. Notably in the C-URICA-E2, the ninth (λ = .52) and 23rd (λ = .62) items showed weak associations. In contrast, in the original, English-language version, the first (λ = .65), sixth (λ = .70), 14th (λ = .69), 23rd (λ = .69), eighth (λ = .68), and 10th (λ = .54) items exhibited weak associations. The 23rd item (“I am preparing to begin a regular exercise group in the next few weeks”) in both versions exhibited a weak association, indicating that this item does not adequately describe the respondent's actual exercise status. Most participants in this study did not convene or begin a new exercise group when they decided to exercise but rather joined an existing exercise group. Most participants in this study “disagreed” with the ninth item (“I could exercise regularly, but I don't plan to”) and stated that it is not that they did not plan to exercise regularly but rather that their situation prevented them from doing so. The weak association exhibited by this item may be due to cultural differences among participants. Taiwanese adults often cannot regularly exercise because of constraints related to time, location, equipment, partners, and weather (Chen et al., 2011). However, the phrase “don't plan to” does not sufficiently describe why people do not exercise, thereby reducing the association of this item.

The standardized factor loadings, AVE, and CR for all of the items in the C-URICA-E2 satisfied the criteria for convergent validity. The square root of AVE of each construct was greater than the correlation coefficient between the construct and other constructs, indicating favorable discriminatory validity. Moreover, the original, English-language version of the URICA-E2 exhibited similar results for convergent validity. Therefore, both the Chinese- and English-language versions of the URICA-E2 exhibited reasonable construct validity.

Cronbach's α values for the C-URICA-E2 ranged between .80 and .92. These results are consistent with those of Reed (1995) and Lerdal et al. (2009), who found that Cronbach's α values for the English- and Norwegian-language versions of the URICA-E2, respectively, ranged from .81 to .94 and .72 to .92. Therefore, the Chinese, English, and Norwegian versions of the URICA-E2 all exhibited excellent internal consistency across each of the six exercise stages of change.

**Figure 2.**
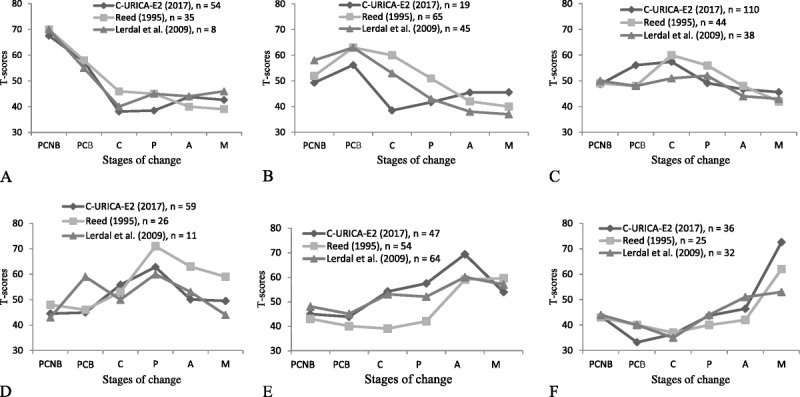
Cluster analysis of the Chinese-language version of the University of Rhode Island Change Assessment-Exercise 2 (C-URICA-E2) and comparison of the results with those of other studies. PCNB = precontemplation-nonbeliever; PCB = precontemplation-believer; C = contemplation; P = preparation; A = action; M = maintenance.

In this study, a cluster analysis was used to group participants into discrete exercise stages. The results were consistent with those of Reed (1995) and Lerdal et al. (2009). Comparing the *T* scores of the six exercise stages of change among the Chinese, English, and Norwegian versions of the URICA-E2 (Figure 2) revealed that the *T* scores of the PCNB stage were similar among the three versions. The *T* scores of the PCB stage for the C-URICA-E2 were lower than those for the English and Norwegian versions. The *T* scores of the contemplation and preparation stages for the original, English version were higher than those for the Chinese and Norwegian versions. In addition, the *T* scores of the action and maintenance stages for the Chinese version were higher than those for the English and Norwegian versions. However, the participant distribution of exercise stages of change varied among these studies. The largest percentage of the 325 participants who completed the Chinese-language version were identified as being in the contemplation stage (33.8%, *n* = 110), whereas the largest percentage of the 249 participants completing the original, English-language version were in the PCB stage (26.1%, *n* = 65), and the largest percentage of the 198 participants who completed the Norwegian version were in the action stage (32.3%, *n* = 64). This uneven distribution of participants among the various exercise stages may be due to differences in ethnicity, environment, and culture among these three studies, indicating that the exercise stages of change are dynamic and vary according to behavioral changes.

This study was affected by two limitations. First, the psychometric analysis targeted adults in a metropolitan area. Consequently, the validity of this instrument for assessing the exercise behaviors of other age groups and of people living in nonmetropolitan areas is unclear. In future studies, the C-URICA-E2 and the associated verification methods should extensively be applied to people in other age groups and in nonmetropolitan areas to increase the applicability of the instrument. Second, a one-time data collection method was adopted. Hence, the applicability of the C-URICA-E2 to determining the evolution of the exercise stages of change (progress, regression, and cessation) over extended periods is unclear. In the future, longitudinal research should be conducted to explore and verify the applicability of this instrument.

### Conclusions

The results of this study, obtained using rigorous procedures, indicate that the Chinese-language version of the URICA-E2 has high content validity, construct validity, internal consistency, and stability. This instrument may be used to continuously assess the exercise stages of change and may enable healthcare professionals to provide appropriate interventions for people at various exercise stages to enhance their health. The findings of this study offer a reference to assist the administrative and academic communities to develop effective exercise intervention policies and solutions for people at various exercise stages of change.
